# Evaluating the Risk of *Clostridioides difficile* Infection After Rifaximin Treatment for Small Intestinal Bacterial Overgrowth

**DOI:** 10.3390/jcm15124449

**Published:** 2026-06-09

**Authors:** Abdelrahman Yousef, Niven Wang, Mahmoud Yousef, Khaled Elfert, Ahmed Telbany, Arman Vaghefi, Kevin Nguyen, Katherine Ripley, Kara Rieth, Daniel Peverini, Fadl Zeineddine, Hareesh K. Gundlapalli, Kaushik Kondubhatla, Abu Baker Sheikh, Archana Kaza, Eliseo F. Castillo, Christopher Chang, Aleksandr Birg

**Affiliations:** 1 Internal Medicine Department, The University of New Mexico Health Sciences Center, Albuquerque, NM 87131, USA; abyousef@salud.unm.edu (A.Y.);; 2 General Medicine Department, SUNY Upstate Medical University, Syracuse, NY 13210, USA; 3 Division of Gastroenterology and Hepatology, West Virgina University, Morgantown, WV 26506, USA; 4 Division of Gastroenterology and Hepatology, The University of New Mexico Health Sciences Center, Albuquerque, NM 87131, USA; 5 Comprehensive Cancer Center, The University of New Mexico Health Sciences Center, Albuquerque, NM 87131, USA; 6 Gastroenterology Department, Raymond G. Murphy Veterans Affairs Medical Center, Albuquerque, NM 87108, USA

**Keywords:** SIBO, small intestinal bacterial overgrowth, Rifaximin, Cdiff, *Clostridioides difficile* infection, irritable bowel syndrome

## Abstract

**Background:** Rifaximin is widely used in the management of small intestinal bacterial overgrowth (SIBO), but concerns remain regarding the potential risk of *Clostridioides difficile* infection (CDI), particularly with repeated antibiotic exposure. **Aim:** To evaluate the short-term risk of CDI following Rifaximin therapy in patients with SIBO. **Materials and Methods:** We conducted a retrospective cohort study using the TriNetX Collaborative Research Network. Adult patients with SIBO were identified and categorized based on Rifaximin exposure within 60 days of diagnosis. The primary analysis compared patients with SIBO treated with Rifaximin to those with SIBO who did not receive Rifaximin. Secondary analyses included comparisons between SIBO patients treated with Rifaximin and irritable bowel syndrome (IBS) patients receiving Rifaximin, as well as patients with SIBO receiving a single versus multiple Rifaximin courses. Propensity score matching (1:1) was performed to balance baseline characteristics. The primary outcome was CDI within 60 days of the index event. Secondary outcomes included hospitalization and emergency department (ED) visits. **Results:** After propensity score matching, 19,597 patients were included in each cohort in the primary comparison of SIBO treated with Rifaximin versus SIBO without Rifaximin. CDI occurred in 0.21% of Rifaximin-treated patients and 0.15% of untreated patients (*p* = 0.152). In the contextual comparison, CDI incidence was similar between SIBO patients receiving Rifaximin and IBS patients receiving Rifaximin (0.21% vs. 0.15%, *p* = 0.168). Among patients with SIBO receiving Rifaximin, CDI risk did not differ between single and multiple treatment courses (0.20% vs. 0.21%, *p* = 0.850). **Conclusions:** In this large real-world cohort, Rifaximin therapy for SIBO was not associated with a statistically significant increase in short-term CDI risk. However, given the low event rate, wide confidence intervals, and risk of type II error, these findings should be interpreted with caution.

## 1. Introduction

The human intestinal microbiota represents a highly complex and dynamic microbial ecosystem. Under normal conditions, the small intestine contains relatively low concentrations of bacteria compared with the colon, with bacterial density increasing progressively toward the distal ileum and large bowel [1,2,3,4,5]. Small intestinal bacterial overgrowth (SIBO) occurs when regulatory mechanisms that maintain microbial balance, such as gastric acid secretion, coordinated intestinal motility, intact anatomy, and mucosal immune defenses, are impaired [6]. Common predisposing conditions include hypochlorhydria, small bowel dysmotility, structural abnormalities such as blind loops or diverticulosis, and immune deficiencies [7]. Bacterial proliferation in the small intestine may provoke mucosal inflammation and structural alterations, including villous blunting and increased intraepithelial lymphocytes, which have been shown to improve following antibiotic therapy [8,9,10].

The clinical presentation of SIBO is variable and frequently reflects malabsorption and fermentation-related symptoms. Patients commonly experience bloating, abdominal distension, diarrhea, and weight loss. Fat malabsorption may lead to deficiencies in fat-soluble vitamins, while vitamin B12 deficiency can result in megaloblastic anemia and neurologic manifestations. Because these symptoms are nonspecific, SIBO is often mistaken for functional gastrointestinal disorders such as diarrhea-predominant irritable bowel syndrome (IBS-D), further complicating diagnosis [11,12,13,14,15].

Management of SIBO involves correction of underlying etiologies when feasible, nutritional optimization, and antimicrobial therapy [16,17]. Historically, systemic antibiotics were employed; however, their broader systemic effects and disruption of colonic microbiota are well recognized. Rifaximin, a minimally absorbed, gut-selective antibiotic, achieves high intraluminal concentrations with limited systemic exposure and has therefore become widely used in SIBO management [18,19,20,21,22]. Reported eradication rates approach 70% in pooled analyses [20]. Nevertheless, recurrence is common, with rates exceeding 40% within nine months in some cohorts, often necessitating repeat antibiotic courses [17,23,24].

Despite its favorable pharmacokinetic profile, concerns persist regarding antibiotic-associated complications, particularly *Clostridioides difficile* infection (CDI). Both SIBO and antibiotic exposure may disrupt microbial homeostasis, theoretically increasing susceptibility to CDI. Moreover, the safety implications of repeated Rifaximin courses in patients with recurrent SIBO remain insufficiently characterized.

In the present study, we examine the risk of CDI among patients with SIBO treated with Rifaximin using a large, multi-institutional federated database. We additionally evaluate whether repeated Rifaximin exposure confers increased short-term CDI risk compared with single-course therapy using a propensity score-matched cohort design.

## 2. Methods

### 2.1. Data Source

For this analysis, we utilized the TriNetX Collaborative Research Network, a federated cloud-based analytics platform that aggregates de-identified electronic health records (EHRs) from more than 80 healthcare organizations across the United States [25,26]. The network provides access to longitudinal clinical data, including demographic characteristics, diagnoses, procedures, medication prescriptions, and laboratory measurements, thereby facilitating large-scale real-world outcomes research.

All data within the TriNetX platform are de-identified in accordance with the Health Insurance Portability and Accountability Act (HIPAA). As the dataset contains no protected health information, this study was exempt from institutional review board review and informed consent requirements. The TriNetX platform incorporates automated quality assurance measures and supports cohort construction, incidence estimation, survival analysis, and propensity score matching [25].

The authors used ChatGPT (OpenAI; GPT-5.5) for language editing and manuscript drafting assistance. All scientific content, analyses, interpretations, and final revisions were reviewed and approved by the authors, who take full responsibility for the manuscript.

### 2.2. Study Design and Cohort Definition

We performed a retrospective cohort study to assess the risk of *Clostridioides difficile* infection (CDI) following Rifaximin therapy in adult patients diagnosed with small intestinal bacterial overgrowth (SIBO). Three complementary analyses were conducted to evaluate the association between Rifaximin exposure and CDI risk.

### 2.3. Primary Analysis: SIBO with Rifaximin vs. SIBO Without Rifaximin

Adult patients (≥18 years) with a documented diagnosis of SIBO (ICD-10 codes K63.821, K63.829, K63.82) were identified within the TriNetX Research Network. The exposed cohort consisted of patients who received Rifaximin (RxNorm 35619) within two months of the SIBO diagnosis. The comparator cohort included patients diagnosed with SIBO who did not receive Rifaximin. This comparison was designed to evaluate whether Rifaximin exposure itself was associated with an increased risk of CDI among patients with SIBO.

### 2.4. Secondary Analysis

#### 2.4.1. SIBO with Rifaximin vs. IBS with Rifaximin

To explore whether the underlying diagnosis of SIBO, which is characterized by alterations in gut microbial composition, modifies the risk of CDI following antibiotic exposure, we conducted a secondary analysis comparing patients with SIBO treated with Rifaximin to adults diagnosed with irritable bowel syndrome (IBS) (ICD-10 K58) who also received Rifaximin. This comparison was designed to assess whether the altered intestinal microbiome associated with SIBO predisposes patients to CDI when exposed to Rifaximin. Individuals with any concurrent SIBO diagnosis were excluded from the IBS cohort to ensure mutually exclusive groups.

#### 2.4.2. Single vs. Multiple Rifaximin Courses in SIBO

Within the SIBO population, we further evaluated whether repeated Rifaximin exposure influenced CDI risk. Adult patients meeting SIBO diagnostic criteria who received Rifaximin within two months of diagnosis were stratified into two groups: (1) a single-course cohort, defined as patients with one recorded Rifaximin prescription; and (2) a multiple-course cohort, defined as patients with two or more recorded Rifaximin prescriptions during the study period.

### 2.5. Exclusion Criteria

Patients younger than 18 years were excluded from all analyses. To minimize confounding from alternative indications for Rifaximin, individuals with diagnoses associated with hepatic encephalopathy or cirrhosis were also excluded. In the IBS comparator cohort, patients with any concurrent diagnosis of SIBO were additionally excluded to ensure mutually exclusive study populations.

### 2.6. Index Event and Outcomes

For rifaximin-treated cohorts, the index date was defined as the date of the first qualifying rifaximin prescription recorded within 60 days of the relevant diagnosis. For the untreated SIBO comparator cohort, the index date was defined as the date of the first qualifying SIBO diagnosis. All outcomes were assessed from the index date forward. This approach was adopted to minimize the risk of immortal-time bias by anchoring the observation window to the treatment decision rather than the diagnostic date. Patients with documented CDI prior to the start of the outcome window were excluded from outcome-specific analyses to ensure inclusion only of individuals at risk. The primary outcome was new-onset *Clostridioides difficile* infection (ICD-10 A04.7) occurring within 60 days following the index event. Secondary outcomes included inpatient hospitalization and emergency department visit. Outcome definitions were based on standardized ICD-10 and visit-type codes within the TriNetX platform.

### 2.7. Statistical Analysis

All analyses were performed using the built-in real-time analytic tools of the TriNetX Live platform (TriNetX LLC, Cambridge, MA, USA). Baseline characteristics were summarized using means with standard deviations for continuous variables and frequencies with percentages for categorical variables. Risk differences, risk ratios, odds ratios, and 95% confidence intervals were calculated for binary outcomes. A two-sided *p*-value < 0.05 was considered statistically significant. All figures and visualizations were generated using Python (version: 3.13.1; Python Software Foundation, Wilmington, DE, USA) using standard scientific computing libraries.

### 2.8. Propensity Score Matching

To mitigate confounding, one-to-one propensity score matching was conducted between comparison cohorts using logistic regression. Matching was performed using a greedy nearest-neighbor algorithm without replacement, applying a caliper width of 0.1 of the pooled standard deviation of the logit of the propensity score. Adequate covariate balance was defined as standardized mean differences (SMD) <0.1 across matched variables.

Covariates incorporated into the propensity score model included: demographics (age, sex, race, ethnicity); comorbid diagnoses (chronic kidney disease, Crohn’s disease, ulcerative colitis, and other gastrointestinal diagnoses); medication utilization (proton pump inhibitors including omeprazole and pantoprazole, H2-receptor antagonists including famotidine, and antacids); and prior healthcare utilization (inpatient hospitalization encounters).

## 3. Results

### 3.1. Study Population

**Figure 1:**
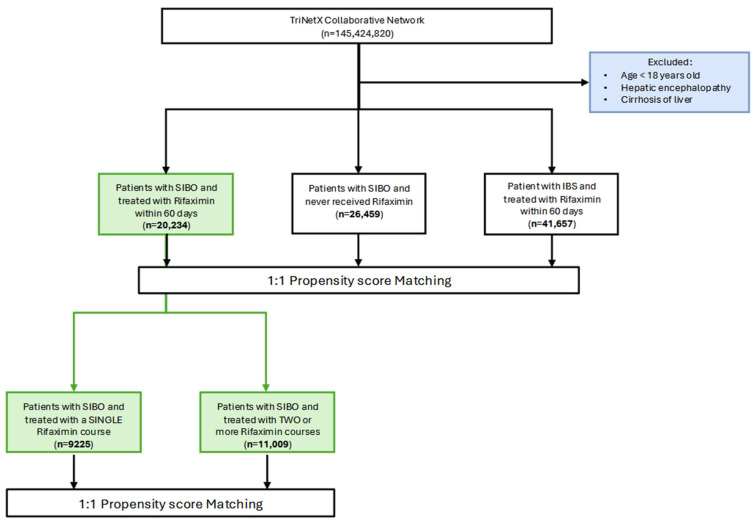
Study cohort selection and design. Flow diagram illustrating cohort selection from the TriNetX Collaborative Research Network. Adult patients with small intestinal bacterial overgrowth (SIBO) were identified and categorized by Rifaximin exposure. Exclusion criteria included age < 18 years, hepatic encephalopathy, and cirrhosis. Primary analysis compared SIBO patients treated with Rifaximin versus those not receiving Rifaximin. A contextual comparison evaluated SIBO patients treated with Rifaximin versus irritable bowel syndrome (IBS) patients treated with Rifaximin. Within the Rifaximin-treated SIBO cohort, a secondary analysis compared single versus multiple Rifaximin courses after propensity score matching.

In the primary analysis, 20,234 patients with SIBO treated with Rifaximin and 26,459 patients with SIBO not treated with Rifaximin were initially identified (Figure 1). After 1:1 propensity score matching, 19,597 patients remained in each cohort. Post-matching baseline characteristics were well balanced, with all standardized mean differences < 0.1. The mean age was 51.0 ± 17.3 years in the Rifaximin cohort and 50.9 ± 17.7 years in the non-Rifaximin cohort (*p* = 0.58). Approximately 75% of patients were female in both groups (*p* = 0.66), and the majority of the patients were white 78.6% vs. 79.3% (*p* = 0.13) (Table 1).

In the secondary analysis, 20,234 patients with SIBO treated with Rifaximin and 41,657 patients with IBS treated with Rifaximin were initially identified. After propensity score matching, 20,228 patients remained in each cohort. Baseline characteristics were well balanced after matching, with all standardized mean differences < 0.1. The mean age was 51.0 ± 17.2 years in the SIBO cohort and 51.1 ± 17.3 years in the IBS cohort (*p* = 0.56).

In the secondary analysis evaluating repeated exposure, 9225 patients with SIBO receiving a single Rifaximin course and 11,009 patients receiving two or more courses were identified. After propensity score matching, 8807 patients remained in each cohort. Baseline characteristics were well balanced following matching, with all standardized mean differences < 0.1. The mean age in both cohorts was 50.8 years (*p* = 0.96), and approximately 75% of patients were female (*p* = 0.72) (Table 2).

Among patients who received two or more courses of Rifaximin, the mean number of Rifaximin prescriptions was 3.55 (SD 4.55), with a median of 2 courses.

### 3.2. Primary Outcome

#### *Clostridioides difficile* Infection

**Figure 2:**
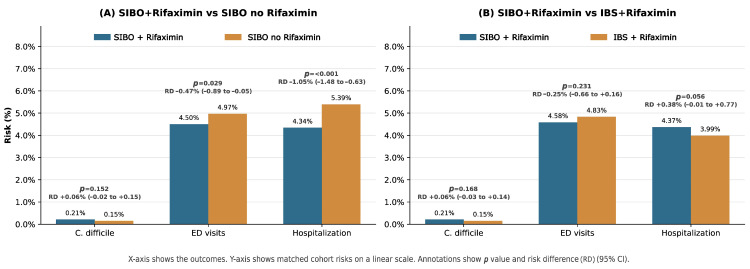
Matched outcome risks across cohort comparisons. Matched risks of *Clostridioides difficile* infection (CDI), emergency department (ED) visits, and hospitalization within 60 days. (**A**) SIBO treated with Rifaximin versus SIBO without Rifaximin. (**B**) SIBO treated with Rifaximin versus IBS treated with Rifaximin. Bars represent absolute risk (%) in each matched cohort; *p*-values indicate between-group comparisons.

In the primary analysis of SIBO patients treated with Rifaximin versus SIBO patients not treated with Rifaximin, CDI within 60 days occurred in 41 of 19,118 patients (0.21%) in the Rifaximin cohort and 29 of 19,099 patients (0.15%) in the non-Rifaximin cohort. There was no statistically significant difference in CDI risk between groups (risk ratio (RR) 1.41; 95% CI 0.88–2.27; *p* = 0.15) (Figure 2).

In the secondary analysis comparing SIBO with Rifaximin versus IBS with Rifaximin, CDI occurred in 42 of 19,725 patients (0.21%) in the SIBO cohort and 30 of 19,555 patients (0.15%) in the IBS cohort. There was no statistically significant difference in CDI risk between groups (RR 1.39; 95% CI 0.87–2.22; *p* = 0.17).

**Figure 3:**
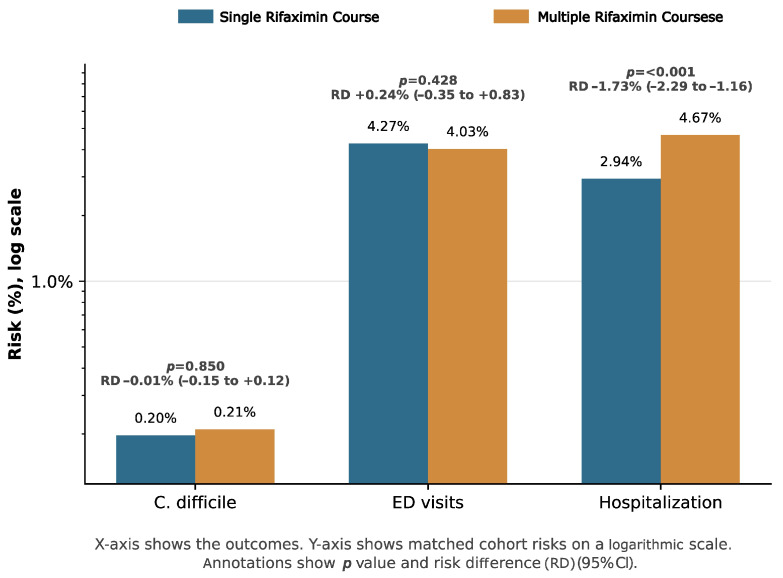
Matched outcomes after single versus multiple Rifaximin courses. Matched risks of *Clostridioides difficile* infection (CDI), emergency department (ED) visits, and hospitalization within 60 days among SIBO patients treated with Rifaximin. Patients receiving a single course were compared with those receiving ≥2 courses after propensity score matching. The *y*-axis is presented on a logarithmic scale.

In the repeated-exposure analysis, CDI within 60 days occurred in 17 of 8629 patients (0.20%) receiving a single Rifaximin course and 18 of 8573 patients (0.21%) receiving two or more courses. There was no statistically significant difference in CDI risk between single-course and multiple-course therapy (RR 0.94; 95% CI 0.48–1.82; *p* = 0.85) (Figure 3).

### 3.3. Secondary Outcomes

#### Hospitalization

In the primary analysis, hospitalization occurred in 851 of 19,597 patients (4.34%) in the SIBO with Rifaximin cohort and 1057 of 19,597 patients (5.39%) in the SIBO without Rifaximin cohort. Hospitalization risk was significantly lower in the Rifaximin-treated group (RR 0.81; 95% CI 0.74–0.88; *p* < 0.01).

In the secondary analysis, hospitalization occurred in 884 of 20,228 patients (4.37%) in the SIBO with Rifaximin cohort and 807 of 20,228 patients (3.99%) in the IBS with Rifaximin cohort. This difference did not reach statistical significance (RR 1.10; 95% CI 1.00–1.20; *p* = 0.06).

In the repeated-exposure analysis, hospitalization occurred in 259 of 8807 patients (2.94%) in the single-course cohort and 411 of 8807 patients (4.67%) in the two-or-more-courses cohort. Hospitalization risk was significantly lower in the single-course cohort (RR 0.63; 95% CI 0.54–0.73; *p* < 0.01).

### 3.4. Emergency Department (ED) Visits

In the primary analysis, emergency department visits occurred in 882 of 19,597 patients (4.50%) in the SIBO with Rifaximin cohort and 974 of 19,597 patients (4.97%) in the SIBO without Rifaximin cohort. ED visit risk was significantly lower in the Rifaximin-treated group (RR 0.91; 95% CI 0.83–0.99; *p* = 0.03).

In the contextual analysis, ED visits occurred in 927 of 20,228 patients (4.58%) in the SIBO with Rifaximin cohort and 978 of 20,228 patients (4.83%) in the IBS with Rifaximin cohort. There was no statistically significant difference between groups (RR 0.95; 95% CI 0.87–1.03; *p* = 0.23).

In the repeated-exposure analysis, ED visits occurred in 376 of 8807 patients (4.27%) in the single-course cohort and 355 of 8807 patients (4.03%) in the two-or-more-courses cohort. There was no statistically significant difference between groups (RR 1.06; 95% CI 0.92–1.22; *p* = 0.43).

## 4. Discussion

In this large, multi-institutional retrospective cohort study, Rifaximin therapy for SIBO was not associated with a statistically significant increase in short-term CDI risk. Across propensity score-matched cohorts, the absolute incidence of CDI within 60 days was low (approximately 0.2%) and did not differ between patients with SIBO treated with Rifaximin and those with SIBO who did not receive Rifaximin. Similarly, CDI risk did not differ between patients with SIBO treated with Rifaximin and those with IBS receiving Rifaximin. Furthermore, among patients with SIBO, repeated Rifaximin exposure was not associated with a statistically significant increased CDI risk compared with a single course of therapy.

Patients with IBS were selected as the matched control group because Rifaximin is an FDA-approved therapy for IBS [27,28,29]. This secondary analysis comparing SIBO and IBS patients receiving rifaximin was designed to evaluate whether the pre-existing microbiome disruption characteristic of SIBO independently modifies CDI susceptibility, independent of antibiotic exposure. While this comparison provides useful context, it is inherently limited by the pathophysiological and clinical differences between these two populations. Despite propensity score matching, residual confounding remains likely, and this analysis should be interpreted as exploratory and hypothesis-generating rather than confirmatory.

Antibiotics remain central to SIBO management, and empiric antibiotic therapy has historically been common in patients with suggestive symptoms and risk factors [6,17]. Exposure to systemic antibiotics is a well-established risk factor for CDI [21,30,31]. Because SIBO involves preexisting alterations in gut microbial composition, concern has existed that antimicrobial therapy, particularly repeated courses, might further predispose patients to CDI. Our data did not demonstrate a statistically significant association in routine clinical practice.

Our results extend prior evidence regarding Rifaximin’s safety profile. A meta-analysis evaluating 17 studies including 815 patients with SIBO treated exclusively with Rifaximin reported an overall adverse event rate of 4.6% (95% CI 2.3–7.5), with only 0.47% of patients discontinuing therapy due to adverse effects [20]. Reported events were generally mild. Notably, only a single case of CDI was described among the included studies, occurring after a four-week course of Rifaximin at 1200 mg daily; details regarding timing and concurrent risk factors were not reported [20,32]. These findings support the low infectious complication rate observed in our cohort.

Consistent with this, clinical trials in SIBO and IBS populations have shown Rifaximin to be well tolerated at doses of 600–1200 mg/day for up to 14 days, with adverse event rates comparable to placebo [33,34,35,36]. Even extended treatment durations have not demonstrated significant drug-related toxicity [37]. Furthermore, clinically meaningful antibiotic resistance has not been widely observed despite more than two decades of use [38], likely reflecting Rifaximin’s mechanism of action involving inhibition of bacterial DNA-dependent RNA polymerase via chromosomal mutation rather than plasmid-mediated resistance pathways [39]. Together, these data reinforce the favorable safety and ecological profile of Rifaximin.

The observed low CDI incidence may be explained by Rifaximin’s pharmacologic properties. Rifaximin is minimally absorbed and achieves high intraluminal concentrations within the gastrointestinal tract while maintaining negligible systemic exposure [18,19,20]. Its limited impact on systemic microbiota and relatively favorable ecological profile may contribute to preservation of colonization resistance against *C. difficile*.

The CDI risk observed with rifaximin in the present study compares favorably to that reported for systemic antibiotics in the broader literature. A large case–control study of over 159,000 community-associated CDI cases, using a comparable real-world claims-based methodology, reported adjusted odds ratios of 25.39 for clindamycin, 9.59 to 12.04 for later-generation cephalosporins, and 6.83 for ciprofloxacin, each relative to no antibiotic exposure [40]. In contrast, the risk ratio observed for rifaximin in our propensity score-matched cohort was non-significant (RR 1.41; 95% CI 0.88–2.27; *p* = 0.15), and the point estimate falls far below the risk levels associated with even the lowest-risk systemic antibiotics in that analysis, further supporting the favorable infectious safety profile of rifaximin in this population. Despite this favorable contextual comparison, these findings should be interpreted with caution given the risk of type II error.

Importantly, recurrence of SIBO is common, with reported rates exceeding 40% within nine months in some studies [23]. As a result, retreatment with Rifaximin is frequently used in clinical practice. Data evaluating the safety of repeated Rifaximin exposure, particularly with respect to CDI risk, have been limited. In our secondary analysis restricted to SIBO patients, multiple Rifaximin prescriptions were not associated with a higher risk of CDI compared with single-course therapy. These findings provide reassurance for clinicians managing recurrent SIBO, where repeated treatment courses are often necessary.

Beyond CDI, we observed differences in healthcare utilization between groups. In the primary comparison of SIBO patients treated with Rifaximin versus those not receiving Rifaximin, hospitalization risk was modestly lower in the Rifaximin-treated cohort. In contrast, hospitalization rates were higher among patients receiving multiple Rifaximin courses compared with those receiving a single course. These secondary outcomes, including hospitalization and emergency department visits, should be interpreted with caution. The lower hospitalization rate observed in rifaximin-treated versus untreated SIBO patients likely reflects differences in baseline disease severity, healthcare access, and unmeasured confounders rather than a direct causal benefit of rifaximin. Similarly, the higher hospitalization rate among patients receiving multiple versus single rifaximin courses likely reflects greater underlying disease burden necessitating repeat treatment. These findings are best regarded as descriptive and hypothesis-generating.

### 4.1. Clinical Implications

From a clinical perspective, these data may help inform antimicrobial stewardship discussions. Although judicious antibiotic use remains essential, our findings suggest that Rifaximin does not confer the same short-term CDI risk signal observed with many systemic antibiotics. For patients with symptomatic SIBO requiring treatment, concerns regarding CDI should not necessarily preclude Rifaximin therapy when clinically indicated.

### 4.2. Limitations

This study has several limitations inherent to retrospective analyses of administrative data. First, the primary outcome (CDI) was ascertained using ICD-10 code A04.7, without laboratory confirmation. While this code has acceptable specificity in administrative data, the sensitivity for mild or outpatient CDI may be limited, and subclinical cases could be underrepresented. This may lead to an underestimate of the true CDI incidence in both groups. Second, the diagnosis of SIBO was ascertained using ICD-10 codes (K63.821, K63.829, K63.82) without confirmation by breath testing or jejunal aspirate culture. This represents a meaningful risk of misclassification bias, as these codes may encompass patients with varying degrees of diagnostic certainty. The absence of standardized diagnostic confirmation limits the interpretability of the findings and their generalizability to populations diagnosed by validated criteria. Third, the 60-day follow-up window was chosen to capture short-term CDI risk attributable to the index antibiotic course. While most antibiotic-associated CDI cases occur within this period, some delayed cases may be missed. The true incidence of rifaximin-associated CDI over longer follow-up periods remains unknown and warrants evaluation in future studies with extended observation windows. Fourth, although propensity score matching reduced measurable confounding across available covariates, residual confounding from unmeasured variables cannot be excluded. Prior antibiotic exposure is one of the strongest predictors of CDI and was not available as a discrete covariate in this analysis. Additionally, markers of SIBO severity, inpatient versus outpatient treatment setting, and functional status were not captured in the TriNetX dataset. These unmeasured factors may differentially affect CDI risk across groups and could bias results in either direction. Fifth, although the overall cohort was large, the absolute number of CDI events was small (41 vs. 29 cases in the primary analysis), substantially limiting statistical power. Accordingly, the risk of a type II error cannot be excluded. These findings should be interpreted in the context of this limitation. Additionally, TriNetX provides de-identified aggregate data, precluding granular review of individual patient records. Finally, no formal pre-specified sensitivity analyses were performed. The TriNetX federated platform imposes constraints on post hoc analytical flexibility, precluding approaches such as E-value calculation or alternative matching ratio analyses. While the consistency of findings across three independent propensity score-matched comparisons provides a degree of analytical triangulation, the absence of formal sensitivity analyses represents a limitation that should be acknowledged when interpreting the robustness of these results.

## 5. Conclusions

In this large, propensity score-matched real-world analysis, Rifaximin therapy in patients with SIBO was associated with a low absolute incidence of CDI and was not associated with a statistically significant increase in short-term CDI risk compared to untreated patients. Additionally, repeated Rifaximin exposure was not associated with statistically significant increased CDI risk compared with a single course. However, results should be interpreted with caution given that the absolute incidence of CDI was low, no statistically significant between-group differences were observed, and the study was limited by a small number of events and wide confidence intervals. Further adequately powered, prospectively designed studies are needed to confirm these findings.

## Figures and Tables

**Table 1:** Baseline characteristics of patients with SIBO treated with Rifaximin compared with patients with SIBO without Rifaximin and IBS with Rifaximin before and after propensity score matching.

	SIBO Treated with Rifaximin vs. SIBO with No Rifaximin						SIBO Treated with Rifaximin vs. IBS Treated with Rifaximin					
	Before Matching			After Matching			Before Matching			After Matching		
Characteristic	SIBO with Rifaximin (*n* = 20,234)	SIBO with No Rifaximin (*n* = 26,455)	*p* Value	SIBO with Rifaximin (*n* = 19,597)	SIBO with No Rifaximin (*n* = 19,597)	*p* Value	SIBO with Rifaximin (*n* = 20,234)	IBS with Rifaximin (*n* = 41,657)	*p* Value	SIBO with Rifaximin (*n* = 20,228)	IBS with Rifaximin (*n* = 20,228)	*p* Value
**Demographics**												
Age at Index	51.0 ± 17.2	51.4 ± 17.6	0.014	51.0 ± 17.3	50.9 ± 17.7	0.576	51.0 ± 17.2	48.6 ± 17.4	<0.001	51.0 ± 17.2	51.1 ± 17.3	0.558
Female	15,096 (74.6%)	20,128 (76.1%)	<0.001	14,662 (74.8%)	14,624 (74.6%)	0.659	15,096 (74.6%)	30,647 (73.6%)	0.007	15,091 (74.6%)	15,405 (76.2%)	<0.001
White	15,918 (78.7%)	20,948 (79.2%)	0.177	15,412 (78.6%)	15,534 (79.3%)	0.131	15,918 (78.7%)	32,834 (78.8%)	0.633	15,917 (78.7%)	16,279 (80.5%)	<0.001
Black or African American	1608 (7.9%)	2107 (8.0%)	0.945	1566 (8.0%)	1521 (7.8%)	0.399	1608 (7.9%)	2761 (6.6%)	<0.001	1607 (7.9%)	1491 (7.4%)	0.030
Hispanic or Latino	1144 (5.7%)	1522 (5.8%)	0.647	1110 (5.7%)	1071 (5.5%)	0.390	1144 (5.7%)	2038 (4.9%)	<0.001	1142 (5.6%)	1023 (5.1%)	0.009
Asian	583 (2.9%)	657 (2.5%)	0.008	552 (2.8%)	494 (2.5%)	0.069	583 (2.9%)	957 (2.3%)	<0.001	579 (2.9%)	533 (2.6%)	0.162
**Diagnosis**												
Chronic kidney disease (CKD)	1509 (7.5%)	1678 (6.3%)	<0.001	1433 (7.3%)	1410 (7.2%)	0.654	1509 (7.5%)	2328 (5.6%)	<0.001	1506 (7.4%)	1352 (6.7%)	0.003
Crohn’s disease (regional enteritis)	1017 (5.0%)	1017 (3.8%)	<0.001	954 (4.9%)	878 (4.5%)	0.069	1017 (5.0%)	2461 (5.9%)	<0.001	1017 (5.0%)	895 (4.4%)	0.004
Ulcerative colitis	698 (3.5%)	637 (2.4%)	<0.001	633 (3.2%)	588 (3.0%)	0.191	698 (3.5%)	1741 (4.2%)	<0.001	698 (3.5%)	554 (2.7%)	<0.001
**Medications**												
Antacids	9588 (47.4%)	9803 (37.1%)	<0.001	9011 (46.0%)	9087 (46.4%)	0.441	9588 (47.4%)	16,939 (40.7%)	<0.001	9582 (47.4%)	9638 (47.6%)	0.577
Omeprazole	7516 (37.1%)	7690 (29.1%)	<0.001	7038 (35.9%)	7139 (36.4%)	0.288	7516 (37.1%)	13,138 (31.5%)	<0.001	7510 (37.1%)	7324 (36.2%)	0.055
Pantoprazole	6313 (31.2%)	6443 (24.4%)	<0.001	5909 (30.2%)	5922 (30.2%)	0.886	6313 (31.2%)	11,746 (28.2%)	<0.001	6311 (31.2%)	6125 (30.3%)	0.045
Famotidine	5849 (28.9%)	6300 (23.8%)	<0.001	5498 (28.1%)	5536 (28.2%)	0.670	5849 (28.9%)	9373 (22.5%)	<0.001	5843 (28.9%)	5697 (28.2%)	0.108
**Encounters**												
Visit: Inpatient Encounter	5850 (28.9%)	8284 (31.3%)	<0.001	5711 (29.1%)	5831 (29.8%)	0.184	5850 (28.9%)	10,957 (26.3%)	<0.001	5847 (28.9%)	5679 (28.1%)	0.064

**Table 2:** Baseline characteristics of patients with SIBO treated with a single Rifaximin course compared with patients treated with two or more Rifaximin courses before and after propensity score matching.

	SIBO Treated with Single vs. Multiple Rifaximin Courses					
	Before Matching			After Matching		
Characteristic	Single Rifaximin Course (*n* = 9225)	Multiple Rifaximin Courses (*n* = 11,009)	*p* Value	Single Rifaximin Course (*n* = 8807)	Multiple Rifaximin Courses (*n* = 8807)	*p* Value
**Demographics**						
Age at Index	50.4 ± 17.6	51.8 ± 16.9	<0.001	50.8 ± 17.5	50.8 ± 17.0	0.964
White	7285 (79.0%)	8633 (78.4%)	0.339	6939 (78.8%)	6982 (79.3%)	0.426
Female	6832 (74.1%)	8264 (75.1%)	0.101	6577 (74.7%)	6556 (74.4%)	0.716
Black or African American	741 (8.0%)	867 (7.9%)	0.681	691 (7.8%)	687 (7.8%)	0.911
Hispanic or Latino	507 (5.5%)	637 (5.8%)	0.373	488 (5.5%)	477 (5.4%)	0.716
Asian	266 (2.9%)	317 (2.9%)	0.986	257 (2.9%)	252 (2.9%)	0.822
**Diagnosis**						
Chronic kidney disease (CKD)	618 (6.7%)	930 (8.4%)	<0.001	614 (7.0%)	611 (6.9%)	0.929
Crohn’s disease (regional enteritis)	343 (3.7%)	689 (6.3%)	<0.001	342 (3.9%)	340 (3.9%)	0.938
Ulcerative colitis	249 (2.7%)	469 (4.3%)	<0.001	248 (2.8%)	225 (2.6%)	0.284
**Medications**						
Antacids	4036 (43.8%)	5797 (52.7%)	<0.001	4030 (45.8%)	4056 (46.1%)	0.694
Omeprazole	3204 (34.7%)	4497 (40.8%)	<0.001	3189 (36.2%)	3175 (36.1%)	0.826
Pantoprazole	2608 (28.3%)	3850 (35.0%)	<0.001	2596 (29.5%)	2638 (30.0%)	0.489
Famotidine	2407 (26.1%)	3622 (32.9%)	<0.001	2403 (27.3%)	2410 (27.4%)	0.906
**Encounters**						
Visit: Inpatient Encounter	2373 (25.7%)	3581 (32.5%)	<0.001	2367 (26.9%)	2400 (27.3%)	0.576

## Data Availability

The data that support the findings of this study are available from the TriNetX Collaborative Research Network. Restrictions apply to the availability of these data, which were used under license for this study and are not publicly available.
